# *Guanxi* beliefs, self-efficacy beliefs, and depressive symptoms—Analysis of the effects of Chinese-style special relationships

**DOI:** 10.3389/fpsyg.2025.1572052

**Published:** 2025-07-11

**Authors:** Guoqing Li, Buerzhasala Ha, Jie Zhang

**Affiliations:** ^1^School of Ethnology and Sociology, Minzu University of China, Beijing, China; ^2^School of Public Health, Shandong University, Jinan, China; ^3^Department of Sociology, State University of New York Buffalo State University, Buffalo, NY, United States

**Keywords:** *guanxi* beliefs, self-efficacy beliefs, depressive symptoms, China, hope, locus of control

## Abstract

**Introduction:**

Depression is a significant global health concern, with a rising number of individuals affected annually. This study examines the influence of cultural and psychological factors on depression within China's collectivist culture, focusing on *guanxi* beliefs (as an external locus of control), self-efficacy beliefs (as an internal locus of control), and the mediating role of hope.

**Methods:**

Using data from the 2018 China Family Panel Studies (CFPS), multivariate linear regression analysis was conducted to explore the relationships between *guanxi* beliefs, self-efficacy beliefs, hope, and depressive symptoms. The sample included 29,225 adults aged 18 and above. Depressive symptoms were measured using an 8-item CES-D scale, while *guanxi* and self-efficacy beliefs were assessed through Likert-scale questions.

**Results:**

Stronger ^*^guanxi^*^ beliefs were associated with increased depressive symptoms (β = 0.206, *p* < 0.001), while stronger self-efficacy beliefs were linked to reduced symptoms (β = −0.151, *p* < 0.001). Hope mediated these relationships, with *guanxi* beliefs reducing hope (β = −0.0129, *p* < 0.01) and self-efficacy enhancing it (β = 0.0867, *p* < 0.001).

**Discussion:**

The findings highlight the distinct roles of cultural (*guanxi*) and psychological (self-efficacy) factors in mental health outcomes. Interventions fostering self-efficacy and hope may mitigate depressive symptoms, particularly in cultures where external factors like *guanxi* are prominent. Future research should explore longitudinal and cross-cultural comparisons to generalize these findings.

## 1 Introduction

### 1.1 Mental health and sense of control

Globally, an increasing number of individuals are enduring the torment of depression. According to the Global Burden of Disease Study (GBD) 2019, the global prevalence of depression has risen significantly from 180 million cases in 1990 to 290 million cases in 2019, marking a substantial increase of 59% (Liu et al., 2024). Based on the statistical data provided by the China Mental Health Survey (CMHS), the lifetime prevalence rate of depressive disorders among adults in China is determined to be 6.8% (Huang et al., 2019). Considering the vast population size of China, the number of individuals corresponding to this prevalence rate is exceedingly substantial.

The etiology of depression is exceedingly intricate, with ongoing research encompassing a spectrum of factors including genetic (Flint and Kendler, 2014; Kendall et al., 2021; Sullivan et al., 2000), biochemical (Gold et al., 2015; Lang and Borgwardt, 2013; Li et al., 2023), and psychosocial (Becker and Kleinman, 2013; Bonde, 2008; Siegrist and Wege, 2020; Zhang et al., 2013, 2018; Zhang and Tao, 2013) dimensions, offering a multifaceted explanation for its origins.

From a macro perspective, a study suggests that depression, as a disease of modernity, is primarily driven by changes in the psychosocial environment (Hidaka, 2012). And the feeling of loss of control over their lives, caused by social factors, is the primary culprit in inducing depression (Ross, 2017). Examples of how cultural factors lead to individuals losing a sense of control over their lives are not uncommon. For instance, traditional patriarchal cultural concepts result in women having limited autonomy over their lives (Bawa, 2012; Davis, 2024; Makama, 2013; Sathar and Kazi, 2022). In collectivist cultures, where family interests are prioritized over individual interests, individuals' choices are restricted (Guess, 2004; LeFebvre and Franke, 2013; Weber and Morris, 2010). Additionally, religious dogmas can also influence individuals' sense of control over their lives (Ellison and Burdette, 2012; Iles-Caven et al., 2020; Schieman, 2008).

In addition to social and cultural factors, individual psychological differences can also affect a person's sense of control over their life, which in turn impacts their mental health. Classic studies in the field of psychology, such as Bandura's self-efficacy (Bandura, 1977) and Seligman's learned helplessness (Maier and Seligman, 1976), are all closely related to the enhancement and loss of a sense of control (Schwarzer, 2014; Shapiro et al., 1996). A reduced sense of self-efficacy correlates with depression (Maddux and Meier, 1995). Repeated experiences of lack of control contribute to learned helplessness (Taylor, 2008), which in turn is the psycho-behavioral model for reactive depression formation (Miller and Seligman, 1975). Enhancing self-efficacy is an effective approach to overcoming learned helplessness (Niknam et al., 2023).

The sense of control is divided into internal and external control. In 1966, Julian B. Rotter introduced the concepts of Internal locus of control (ILOC) and external locus of control (ELOC). Internal locus of control refers to the belief that an individual's behavior and decisions are the primary factors influencing the outcomes in their life, while external locus of control refers to the belief that external factors, such as luck or the actions of others, are the main determinants of the outcomes in their life (Rotter, 1966). External locus of control is highly correlated with depressive symptoms, with an increase in external control points leading to increased depressive symptoms (Benassi et al., 1988; Khumalo and Plattner, 2019; Presson and Benassi, 1996; Yu and Fan, 2014). Internal locus of control, on the other hand, reduces depressive symptoms (Ade and Asep, 2016; Jaswal et al., 1997).

### 1.2 *Guanxi* beliefs and self-efficacy beliefs vs. ELOC and ILOC

We hypothesize that *guanxi* beliefs, as an external locus of control, will significantly influence depressive symptoms. *Guanxi* is a term used in Chinese culture to describe an individual's social network of mutually beneficial personal and business relationships. There is no exact English equivalent for *guanxi*, as it encompasses not only personal relationships and ties but also a form of social capital. *Guanxi* represents a distinctive Chinese approach to social resource allocation (Chen et al., 2013; Lin, 2001). Due to its opaque operational nature, acquiring *guanxi* capital requires initial endowments and luck, making access relatively constrained (Bian, 2019). This aligns with the psychological concept of external locus of control, where individuals perceive life outcomes as determined by external forces rather than personal efforts. The uniqueness of *guanxi* beliefs lies in their cultural embeddedness—unlike generalized external control beliefs, *guanxi* operates within a collectivist framework where relational interdependence is both normative and institutionalized. Thus, *guanxi* beliefs reflect not just perceived lack of control but also a culturally specific mechanism of resource dependence that reinforces external attribution of life outcomes.

While Western research often examines *guanxi* in business contexts (Chen and Chen, 2004; Dunfee and Warren, 2001; Fock and Woo, 1998; Lee et al., 2001), this narrow focus overlooks its pervasive societal role. Guanxi influences all aspects of Chinese society, from village-level concepts like face (*mianzi*) and reciprocity (*renqing*; Yan, 1996) to labor market dynamics (Bian, 2022; DiTomaso and Bian, 2018; Huang, 2008; Zhang and Li, 2003) and policy implementation (Zhan et al., 2014). In China, people widely believe *guanxi* significantly impacts social life (Yeung and Tung, 1996). Previous studies have primarily analyzed *guanxi* as a social network, examining its structures and operations (Bian, 2019; Fan, 2002; Luo, 1997), with less attention to its psychological effects on individuals. We posit that when individuals perceive *guanxi* as crucial for social success, it functions as an external locus of control, diminishing their sense of personal agency over life outcomes and fostering pessimistic future expectations. Therefore, we propose that stronger *guanxi* beliefs will be associated with more severe depressive symptoms.

**Hypothesis 1**.*The stronger the guanxi beliefs, the more severe the depressive symptoms*.

We hypothesize that self-efficacy beliefs, as an internal locus of control, will significantly influence depressive symptoms. It is widely recognized that Chinese people are extremely hardworking (Ruble and Zhang, 2013; Smith, 1894). Behind this diligence lies the Confucian concept of *tian dao chou qin* (heaven rewards diligence), which means that as long as one makes an effort, they will receive corresponding rewards. In this sense, Chinese people value hard work and believe that their efforts can achieve the desired goals, thereby possessing a strong sense of self-efficacy. One who believes that personal effort is important shows a strong identification with an internal locus of control. However, in collectivist cultures (e.g., East Asia), high self-efficacy may be associated with “excessive effort” or “social comparison pressure,” thereby increasing the risk of depression. Research found that in cultures such as Japan, excessively high self-efficacy may conflict with societal expectations, leading to psychological distress (Uchida et al., 2004). To examine the relationship between self-efficacy and depressive symptoms in Chinese society, we propose the following research hypotheses:

**Hypothesis 2**. *The stronger the self-efficacy beliefs, the weaker the depressive symptoms*.

### 1.3 Hope as a mental health protector

Previous studies have found that hope is a protective factor for mental health (Bai et al., 2017; Leite et al., 2019; Snyder et al., 2001; Thimm et al., 2013; Valle et al., 2006). In terms of hope and sense of control, researchers indicate that the lack of hopefulness is related to the perception that external factors control one's life (Brackney and Westman, 1992), while hope helps to strengthen the internal locus of control (Winarsunu et al., 2023). Therefore, we aim to verify whether hope is a mediating variable through which *guanxi* beliefs and self-efficacy beliefs influence depression. We propose the following two hypotheses:

**Hypothesis 3**. *Guanxi beliefs affect depressive symptoms by influencing hope*.
**Hypothesis 4**. *Self-efficacy beliefs affect depressive symptoms by influencing hope*.

## 2 Methods

### 2.1 Data source and analysis methods

The data used in this study come from the 2018 China Family Panel Studies (CFPS), a panel data collection project managed by Peking University in China. CFPS focuses on the economic and non-economic welfare of Chinese residents, as well as many research topics including economic activities, educational achievements, family relations and family dynamics, population migration, health, etc. it is a national, large-scale, multi-disciplinary social tracking survey project. CFPS samples cover 25 provinces/cities/autonomous regions, and the target sample size is 16,000 households. The respondents include all family members in the sample households. The individual dataset includes data from 37,354 respondents aged 10 and above, of which 33,973 are adults.

Since the dependent variable is a continuous variable, multivariate linear regression is used for estimation. We analyzed the data using StataMP 17 statistical software.

### 2.2 Measurements

#### 2.2.1 Dependent variable

The dependent variable in this paper is the depressive symptoms score. In the questionnaire of CFPS2018, the simplified Center for Epidemiological Studies-Depression (CES-D) scale was used to measure depressive symptoms, including 8 related questions such as feeling down, struggling to do things, and feeling lonely. Specifically, the respondents who had the following depressive symptoms in the past week were classified into almost none (< 1 day), sometimes (1–2 days), often (3–4 days), and mostly (5–7 days) according to the frequency, and were assigned scores of 1 to 4 respectively: (1) “I feel down,” (2) “I find it difficult to do anything,” (3) “My sleep is not good,” (4) “I feel happy,” (5) “I feel lonely,” (6) “I live a happy life,” (7) “I feel sad and upset,” (8) “I feel like life can't continue.” In this study, items (4) and (6), which reflect positive emotions, are reverse-scored, and the scores are summed up to obtain a depressive symptoms score. A higher score indicates a more severe depressive symptoms status. The possible range for our dependent variable's score is from 4 (lowest) to 32 (highest).

#### 2.2.2 Independent variables

This article examines the impact of two independent variables on the dependent variable, namely, *guanxi* beliefs and self-efficacy beliefs.

Regarding *guanxi* beliefs, we selected the degree of agreement with the statement “In today's society, having social *guanxi* is more important than individual abilities” from the CFPS2018 questionnaire. The degree of agreement is scored on a scale of 1–5, with 1 indicating “strongly disagree,” 2 indicating “disagree,” 3 indicating “agree,” 4 indicating “strongly agree,” and 5 indicating “neither agree nor disagree.” In this regard, scores were recorded. A score of 1 indicates “strongly disagree,” 2 indicates “disagree,” 3 indicates “neither agree nor disagree,” 4 indicates “agree,” and 5 indicates “strongly agree.” The higher the score, the stronger the agreement with this viewpoint.

Similarly, regarding self-efficacy beliefs, we selected the level of agreement with the statement “In today's society, hard work can be rewarded” from the CFPS2018 questionnaire. The level of agreement was scored on a scale of 1–5, with 1 indicating “strongly disagree,” 2 indicating “disagree,” 3 indicating “agree,” 4 indicating “strongly agree,” and 5 indicating “neither agree nor disagree.” We recoded this, with 1 representing “strongly disagree,” 2 representing “disagree,” 3 representing “neither agree nor disagree,” 4 representing “agree,” and 5 representing “strongly agree.” The higher the score, the stronger the agreement with this viewpoint.

#### 2.2.3 Mediating variables

In order to reveal the mechanism of the influence of *guanxi* beliefs and self-efficacy beliefs on depressive symptoms, we introduce the mediating variable: the degree of confidence in one's future. The mediating variable is selected from the question “How confident are you in your future?” in the CFPS2018 questionnaire. The answers are scored from 1 to 5, with 1 indicating “very little confidence” and 5 indicating “very much confidence.” The higher the score, the higher the confidence.

#### 2.2.4 Control variables

To minimize errors arising from excluding variables during model estimation as much as possible, we incorporated additional pertinent control variables based on prior research findings. The control variables in this article included the following: Individual's gender (female = 0; male = 1), age, marital status (unmarried = 0; married = 1; cohabiting = 2; divorced = 3; widowed =4), years of education, logarithm of income, self-rated health (1–5), *hukou* (rural = 0; urban = 1), life satisfaction (1–5) and popularity (0–10).

Since the *guanxi* awareness of minors is relatively weak and has less impact on them, this article selects adults aged 18 and above as our research subjects.

The impact of marital status on depressive symptoms is multifaceted. The binary classification of marital status (married/unmarried) is conceptually inadequate for sophisticated analyses of its association with depressive symptoms. Therefore, unlike previous studies that simply divided marital status into two categories of married and unmarried, we divided marital status into five categories based on the questionnaire: “unmarried,” “married,” “cohabiting,” “divorced,” and “widowed.”

Regarding years of education, the options provided in the questionnaire include “illiterate/semi-illiterate,” “elementary school,” “junior high school,” “high school/technical school/vocational high school,” “associate degree,” “undergraduate degree,” “master's degree,” “doctoral degree.” In China, in most cases, they correspond to education years of 0, 6, 9, 12, 15, 16, 19, and 23 years, respectively.

The variable of income used in this article is the logarithm of annual working income. The reason for taking logarithms is that the income gap of the sample is relatively large.

The *hukou* system in China, which is a population management system, is divided into rural *hukou* and urban *hukou*. Due to the existing disparities in such as living environments, economic incomes, lifestyles, and educational levels between individuals under the two *hukou* systems, the depression status of these two groups also differs accordingly (Wu et al., 2019). Therefore, we included *hukou* as a variable within our set of control variables in order to reduce the interference from the factor of differing regions.

## 3 Results

### 3.1 Descriptive statistical analysis

Table 1 presents the basic information of the variables in our study. In our sample, there are 14,711 females (50.34%) and 14,514 males (49.66%), with a minimum age of 18 and a maximum age of 96, and an average age of 47. Among the marital status, 3,405 people (11.65%) are unmarried, 23,448 people (80.23%) are married, 128 people (0.44%) are cohabiting, 599 people (2.05%) are divorced, and 1,645 people (5.63%) are widowed. Regarding the years of education, the shortest is 0 years, which is in the state of illiteracy, and the longest is 23 years, which is equivalent to completing a doctoral degree. The average years of education is 7.66 years. With the popularization of the 9-year compulsory education system in China, 7.66 years indicates that it is getting closer to the requirements of the 9-year compulsory education system. The average value of the logarithm of annual income is 3.47. The average self-rated health status is 3. The number of people with rural *hukou* was 21,521, accounting for 73.64%, while the number of people with urban *hukou* was 7,704, accounting for 26.36%. The level of people's life satisfaction is at a high level, with an average of 4.01 out of 5. Similarly, the popularity is also relatively high, with an average value of 7.13 out of 10.

**Table 1:** Variable descriptive statistical analysis (*N* = 29,225).

**Variable**	**Variable definition**	**Freq./Mean**	**Percent (%)/SD**
**DV**			
Depressive symptoms score (8–32)	It involves 8 CESD variables, with a minimum value of 1 and a maximum value of 4 for each variable. After adding up, the minimum value is 8 and the maximum value is 32. Higher scores indicate greater depressive symptoms	13.59	4.01
**IV**			
*Guanxi* beliefs (1–5)	The rating scale of 1 (strongly disagree) to 5 (strongly agree) applies to the viewpoint that *guanxi* matters more than personal effort	3.57	1.00
Self-efficacy beliefs (1–5)	The rating scale of 1 (strongly disagree) to 5 (strongly agree) applies to the viewpoint that hard work brings rewards	4.00	0.79
**MV**			
Hope (1–5)	The corresponding viewpoint in the questionnaire is: confidence in one's own future. The rating scale of 1 (representing very little confidence) to 5 (representing great confidence) applies to one's outlook on their own future	4.13	0.96
**CV**			
**Gender**			
Female (0)		14,711	50.34
Male (1)		14,514	49.66
Age (18–96)	The minimum age is 18, and the maximum age is 96	47.28	16.38
**Marital status**			
Unmarried (0)		3,405	11.65
Married (1)		23,448	80.23
Cohabiting (2)		128	0.44
Divorced (3)		599	2.05
Widowed (4)		1,645	5.63
Years of education	The minimum education period is 0 years, and the maximum education period is 23 years	7.66	5.05
Logarithm of income	The logarithm of annual work income	3.47	4.88
Self-rated health (1–5)	Assign values of 1–5 from “unhealthy” to “very healthy”	3.00	1.22
***Hukou***			
Rural		21,521	73.64
Urban		7,704	26.36
Life satisfaction (1–5)	Assign values of 1–5 from “very dissatisfied” to “very satisfied”	4.01	0.96
Popularity (0–10)	Assign values of 0–10 from “very unpopular” to “very popular”	7.13	1.95

Among the two variables of *guanxi* beliefs and self-efficacy beliefs, a score of 1 represents “strongly disagree” and a score of 5 represents “strongly agree.” The average score for *guanxi* beliefs is 3.57, and the average score for self-efficacy beliefs is 4. This suggests that, in this sample, people not only perceive *guanxi* as important in today's society but also neither deny nor dispute the importance of personal effort, and in many cases, strongly agree with it. In our sample, the lowest score for depressive symptoms is 8, the highest is 32, and the average score is 13.59, indicating that the overall level of depression is not high.

### 3.2 Regression analysis

Table 2 presents the correlation between *guanxi* beliefs, self-efficacy beliefs, and depressive symptoms score in the absence of any control variables. Specifically, the *p*-value associated with the relationship between *guanxi* beliefs and depressive symptoms is statistically significant. Similarly, the *p-*value for the relationship between self-efficacy beliefs and depressive symptoms is also statistically significant. This indicates that both *guanxi* beliefs and self-efficacy beliefs have an impact on the depressive symptoms score.

**Table 2:** Bi-variate analyses of depressive symptoms score with *Guanxi* belief and self-efficacy.

**Dependent variable**	***Guanxi* beliefs**	**Self-efficacy beliefs**
Depressive symptoms score	*r* = 0.0644, *p* < 0.001	*r* = −0.0324, *p* < 0.001

r, Spearman's rank correlation coefficient.

In Table 3, we employed multiple regression analysis to investigate whether the significance of the relationship between *guanxi* beliefs and depressive symptoms score, as well as the relationship between self-efficacy beliefs and depressive symptoms score, persisted when control variables were included. Model 1 incorporates these control variables into the analysis, and upon examining the model fitting results, the following conclusions are drawn. gender, age, years of education, health condition, *hukou*, life satisfaction, popularity all had a significant relationship with the depressive symptoms score. The relationship between marital status and depressive symptoms score is more complex. Compared to being unmarried, there is no significant correlation between being married and depressive symptoms score, but there is a significant correlation between cohabitation, divorce, and widowhood and depressive symptoms score. Women are more prone to depression than men, and individuals with rural *hukou* are more likely to suffer from depression than those with urban *hukou*. Compared to being unmarried, people who are cohabiting, divorced, or widowed are more susceptible to depression. Older age, higher education levels, better health status, higher life satisfaction, and better interpersonal relationships are associated with a lower likelihood of depression. There was no significant correlation between the logarithm of income and depressive symptoms score.

**Table 3:** Multiple regressions with depressive symptoms score as their dependent variables (*N* = 29,225).

**Variables**	**Model 1**	**Model 2**	**Model 3**
Gender (male)	−0.503^***^	−0.520^***^	−0.497^***^
	(−11.47)	(−11.85)	(−11.33)
Age	−0.0211^***^	−0.0206^***^	−0.0208^***^
	(−11.19)	(−10.89)	(−11.01)
**Marital status (reference group: unmarried)**			
Married	−0.00423	−0.0495	−0.00395
	(−0.06)	(−0.65)	(−0.05)
Cohabiting	0.714^*^	0.680^*^	0.695^*^
	(2.19)	(2.09)	(2.13)
Divorced	1.185^***^	1.151^***^	1.188^***^
	(7.26)	(7.06)	(7.28)
Widowed	1.810^***^	1.762^***^	1.803^***^
	(14.08)	(13.72)	(14.03)
Years of education	−0.111^***^	−0.110^***^	−0.113^***^
	(−20.28)	(−20.03)	(−20.56)
Logarithm of income	−0.00207	−0.00308	−0.00283
	(−0.42)	(−0.63)	(−0.58)
Self-rated health	−0.896^***^	−0.893^***^	−0.890^***^
	(−47.71)	(−47.64)	(−47.33)
*Hukou* (urban)	−0.497^***^	−0.505^***^	−0.521^***^
	(−9.35)	(−9.52)	(−9.77)
Life satisfaction	−0.902^***^	−0.898^***^	−0.890^***^
	(−38.98)	(−38.86)	(−38.33)
Popularity	−0.126^***^	−0.126^***^	−0.123^***^
	(−11.31)	(−11.32)	(−11.02)
*Guanxi* beliefs		0.206^***^	
		(9.83)	
Self-efficacy beliefs			−0.151^***^
			(−5.53)
F	601.81	564.77	558.42
*P*	<0.001	<0.001	<0.001
*R* ^2^	0.1982	0.2009	0.1991

* *p* < 0.05,

*** *p* < 0.001.

In Model 2, we introduce the first independent variable: *guanxi* beliefs. The regression results show that there is still a significant correlation between *guanxi* beliefs and depressive symptoms score. We can see from Model 2 that every 1-point increase in *guanxi* beliefs is associated with a corresponding 0.206-point increase in depressive symptoms score, indicating that the more people believe that *guanxi* play an important role in today's society, the more likely they are to become depressed. In Model 3, we introduce a second independent variable, self-efficacy beliefs, to observe its impact on depressive symptoms score. The regression results show that there is still a significant relationship between the two. We can see from the model 3 that for every 1-point increase in self-efficacy beliefs, the depressive symptoms score decreases by 0.151 points. This suggests that the more one believes in the usefulness of personal effort, the less likely they are to become depressed.

To reveal the mechanism of the influence of two beliefs and depressive symptoms score, we introduced the mediating variable: hope. As shown in Table 4, we introduced the mediating variable hope into models 4 and 5 to examine the impact of *guanxi* beliefs and self-efficacy beliefs on the dependent variable depressive symptoms score. We can see that in both models 4 and 5, hope is significantly correlated with depressive symptoms score. In Model 4, for every 1-point increase in hope, the depressive symptoms score decreases by 0.494 points. In Model 5, for every 1-point increase in hope, the depressive symptoms score decreases by 0.489 points. From model 2 to model 4, the estimated coefficient of *guanxi* beliefs on depressive symptoms score decreased from 0.206-point to 0.200-point. From Model 3 to Model 5, the estimated coefficient of self-efficacy beliefs on depressive symptoms score increased from −0.151 points to −0.108 points. Therefore, hope can explain the partial influence of *guanxi* beliefs and self-efficacy beliefs on the dependent variable depressive symptoms score.

**Table 4:** Regression estimation results of mediating variable on depressive symptoms score (*N* = 29,225).

**Variables**	**Model 4**	**Model 5**
Gender (male)	−0.501^***^	−0.481^***^
	(−11.49)	(−11.02)
Age	−0.0234^***^	−0.0237^***^
	(−12.45)	(−12.58)
**Marital status (reference group: unmarried)**		
Married	0.0186	0.0620
	(0.24)	(0.82)
Cohabiting	0.718^*^	0.737^*^
	(2.22)	(2.28)
Divorced	1.217^***^	1.251^***^
	(7.51)	(7.72)
Widowed	1.790^***^	1.832^***^
	(14.02)	(14.34)
Years of education	−0.110^***^	−0.113^***^
	(−20.21)	(−20.64)
Logarithm of income	−0.00248	−0.00205
	(−0.51)	(−0.42)
Self-rated health	−0.849^***^	−0.848^***^
	(−45.23)	(−45.06)
*Hukou* (Urban)	−0.529^***^	−0.538^***^
	(−10.03)	(−10.15)
Life satisfaction	−0.675^***^	−0.673^***^
	(−26.27)	(−26.11)
Popularity	−0.0938^***^	−0.0919^***^
	(−8.39)	(−8.20)
*Guanxi* beliefs	0.200^***^	
	(9.59)	
Hope	−0.494^***^	−0.489^***^
	(−19.19)	(−18.93)
Self-efficacy beliefs		−0.108^***^
		(−3.98)
F	557.34	550.47
*P*	<0.001	<0.001
R^2^	21.08	20.88

* *p* < 0.05,

*** *p* < 0.001.

**Figure 1:**
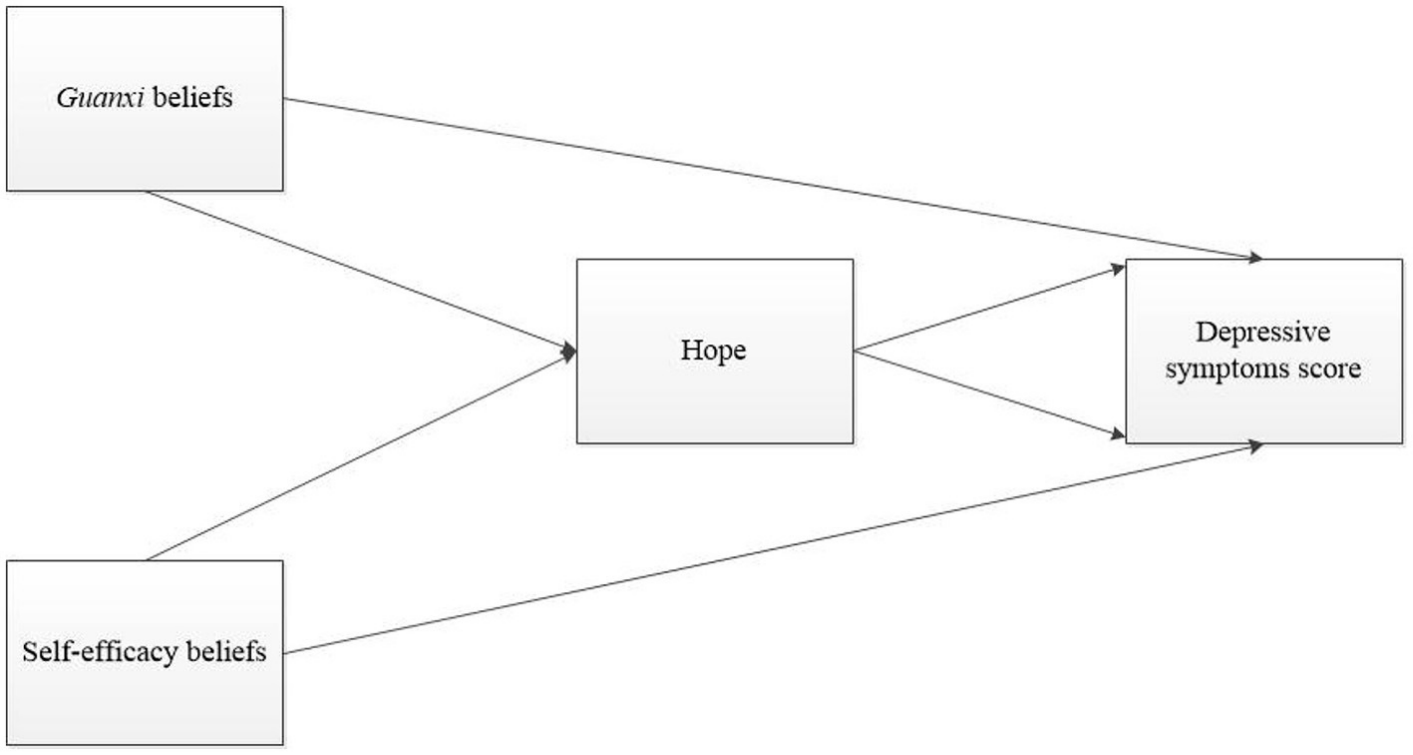
The analysis model of the relationship between guanxi beliefs, self-efficacy beliefs, hope, and depressive symptoms score.

To confirm whether hope is the mediating mechanism of two beliefs and depressive symptoms score (Figure 1), we further examined the relationship between two beliefs and hope. The results are shown in Table 5. As can be seen from Model 6, there is a significant correlation between *guanxi* beliefs and hope (*p* < 0.01). For every 1 point increase in *guanxi* beliefs, hope decreases by 0.0129 points. As can be seen from Model 7, there is a significant correlation between self-efficacy beliefs and hope (*p* < 0.001). For every 1-point increase in self-efficacy beliefs, hope increases by 0.0867 points.

**Table 5:** Regression estimation results of the relationship between dependent variables and mediating variable (*N* = 29,225).

**Variables**	**Model 6 Hope**	**Model 7 Hope**
Gender (male)	0.0377^***^	0.0330^***^
	(3.80)	(3.34)
Age	−0.00579^***^	−0.00595^***^
	(−13.58)	(−13.99)
**Marital status (reference group: unmarried)**		
Married	0.138^***^	0.135^***^
	(7.98)	(7.84)
Cohabiting	0.0764	0.0851
	(1.04)	(1.16)
Divorced	0.134^***^	0.131^***^
	(3.64)	(3.56)
Widowed	0.0570^*^	0.0577^*^
	(1.96)	(2.00)
Years of education	−0.000528	0.000518
	(−0.43)	(0.42)
Logarithm of income	0.00122	0.00159
	(1.11)	(1.45)
Self-rated health	0.0890^***^	0.0857^***^
	(21.01)	(20.26)
*Hukou* (Urban)	−0.0485^***^	−0.0356^**^
	(−4.05)	(−2.97)
Life satisfaction	0.450^***^	0.444^***^
	(86.23)	(84.92)
Popularity	0.0650^***^	0.0632^***^
	(25.88)	(25.22)
*Guanxi* beliefs	−0.0129^**^	
	(−2.71)	
Self-efficacy beliefs		0.0867^***^
		(14.13)
F	883.99	904.59
*P*	<0.001	<0.001
*R* ^2^	28.23	28.70

* *p* < 0.05,

** *p* < 0.01,

*** *p* < 0.001.

**Figure 2:**
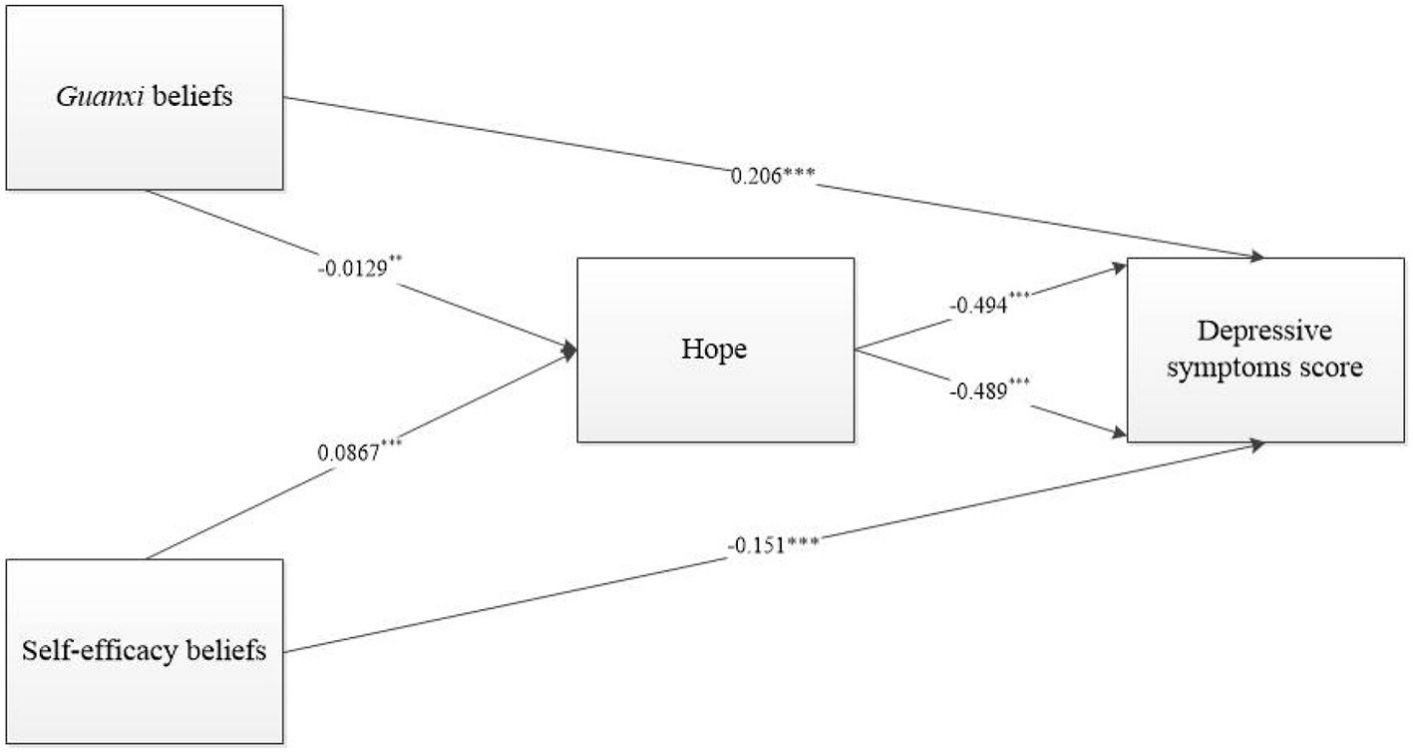
Model of mediating effects of hope. ***p < 0.001 **p < 0.01.

It can be seen that *guanxi* beliefs reduce people's hope for the future, thereby increasing their level of depressive symptoms. In contrast, self-efficacy beliefs enhance people's hope for the future, thereby reducing their level of depressive symptoms (Figure 2).

## 4 Discussion and conclusion

*Guanxi*, as a form of external locus of control, has an omnipresent influence on Chinese people. The Chinese attitude toward *guanxi* is extremely complex. For Chinese people, *guanxi* is both sensitive and reasonable. The sensitivity arises because the existence of *guanxi* inevitably leads to unfairness. Whether it is others gaining benefits through *guanxi* at the expense of one's own rights, or oneself gaining benefits through *guanxi* at the expense of others' rights, it touches upon the issue of fairness and justice in resource allocation. This causes people to become immediately tense when they mention or hear about *guanxi*. The reasonableness is acknowledged because *guanxi* has always been and is densely present in society, and it is difficult to break its existence. To a certain extent, it represents a form of power, so people have to accept the legitimacy of *guanxi*.

Previous studies either analyzed the operation mode of *guanxi* in the Chinese market from a business perspective, or treated *guanxi* as a social network or ethical system, conducting structural decomposition and analysis, neglecting the impact of *guanxi* on individual feelings and the psychological effects these feelings have on them. This study aims to fill this gap by discovering the impact of *guanxi* beliefs on depressive symptoms among Chinese people. At the same time, we also pay attention to self-efficacy, which corresponds to the internal locus of control, and its important role in the mental health of Chinese people, thus making up for the shortcomings of previous research in this regard. Finally, we verified whether hope, as a mediating factor, plays a mediating role in the impact of *guanxi* beliefs and self-efficacy beliefs on depressive symptoms. Finally, we reached the following conclusions:

Firstly, our results support the hypothesis that stronger *guanxi* beliefs are associated with more severe depressive symptoms. This relationship is consistent with the notion that an external locus of control, which is heightened by the importance placed on *guanxi*, can lead to a diminished sense of personal control over one's life outcomes. Consequently, this can result in increased feelings of helplessness and depression.

Secondly, the study confirms that stronger self-efficacy beliefs are associated with weaker depressive symptoms. This aligns with the internal locus of control, where individuals who believe in their ability to influence their life outcomes through personal effort exhibit lower levels of depression. This finding underscores the protective role of self-efficacy in mental health.

Thirdly, our research reveals that hope acts as a significant mediator in the relationship between both *guanxi* beliefs and self-efficacy beliefs with depressive symptoms. The mediating effect of hope suggests that the influence of cultural beliefs and personal agency on mental health is partially explained by the level of optimism and confidence in one's future. Specifically, *guanxi* beliefs reduce hope, thereby increasing depressive symptoms, while self-efficacy enhances hope and reduces the likelihood of depression.

The innovation of our research lies in the discovery that *guanxi*, as a cultural factor, indeed has an impact on the mental health of Chinese people. It is worth noting that this perception of *guanxi* is highly subjective. That is to say, if one believes that *guanxi* plays a significant role in the allocation of social resources, then their level of depression will increase. In contrast, self-efficacy, as a form of psychological energy, has a positive effect on individual mental health. In other words, the more one believes in the importance of personal effort, the weaker their depressive symptoms will be. Therefore, by introducing mediating variables, this paper suggests that part of the reason for the above two patterns is the different levels of hope that individuals gain. That is, when a person has a strong belief in *guanxi*, it will reduce their sense of hope, thereby exacerbating the degree of depression. On the contrary, when a person has a strong belief in self-efficacy, it will increase their sense of hope, thereby reducing the degree of depression.

While we agree that multi-item scales would provide richer data, the CFPS—as a nationally representative longitudinal survey—prioritizes breadth over depth in construct measurement. Our single-item approach aligns with its trade-off strategy, and its significant correlation with depression scores supports convergent validity. We caution against overgeneralizing the results but argue its appropriateness for population-level analysis.

The findings suggest that mental health interventions in China should prioritize fostering self-efficacy and hope while addressing the psychological impacts of *guanxi* beliefs. In practice, on the one hand, education should be employed to raise awareness of the significance of self-efficacy and enhance individuals' trust in their own capabilities. On the other hand, it is necessary to promote equitable social development, mitigate the pervasive sense of unfairness among people, and diminish the influence of social *guanxi* in resource allocation, thereby creating a social environment where individuals can achieve success based on their personal abilities rather than relying on social *guanxi*.

While this exploratory study provides valuable insights, several limitations should be acknowledged. First, the study employed a parallel predictor analysis to examine the independent effects of guanxi beliefs and self-efficacy on depressive symptoms but did not compare their relative effect sizes—future research could adopt multigroup structural equation modeling (SEM) or effect size comparison for a more systematic assessment. Second, due to the cross-sectional design, causal inferences remain speculative; longitudinal or experimental studies are needed to clarify directional relationships. Third, depressive symptoms were measured using an abbreviated 8-item CES-D (adopted by CFPS in 2016–2018), which, though validated for screening (see the CFPS-39 Technical Report), may lack the sensitivity of the full 20-item version. Lastly, the absence of cross-cultural comparisons (e.g., with East Asian societies like Japan or Korea) limits generalizability—future work could explore cultural variations in these associations. These limitations highlight avenues for refining theoretical models, measurement tools, and comparative frameworks.

In conclusion, this study provides empirical evidence that cultural beliefs in *guanxi* and psychological beliefs in self-efficacy have distinct impacts on depressive symptoms in China, with hope being a crucial mediator in these relationships. These findings have important implications for mental health policies and interventions. They suggest that fostering a sense of self-efficacy and hope may be beneficial in reducing depressive symptoms, particularly in cultures where external factors like *guanxi* are highly valued. Future research should further explore the dynamic interplay between cultural beliefs, psychological factors, and mental health, and how these relationships might be influenced by societal changes and economic development.

## Data Availability

The original contributions presented in the study are included in the article/supplementary material, further inquiries can be directed to the corresponding author.
